# Analyzing postoperative complications in colorectal cancer surgery: a systematic review enhanced by artificial intelligence

**DOI:** 10.3389/fsurg.2024.1452223

**Published:** 2024-10-31

**Authors:** Dan Andras, Angela Madalina Lazar, Dragoş Crețoiu, Florian Berghea, Dragos Eugen Georgescu, Valentin Grigorean, Simona Raluca Iacoban, Bogdan Mastalier

**Affiliations:** ^1^ Colentina General Surgery Clinic, Carol Davila University of Medicine and Pharmacy, Bucharest, Romania; ^2^ General Surgery Clinic, Colentina Clinical Hospital, Bucharest, Romania; ^3^ Department of Genetics, Carol Davila University of Medicine and Pharmacy, Bucharest, Romania; ^4^ Fetal Medicine Excellence Research Center, Alessandrescu Rusescu National Institute for Maternal and Child Health, Bucharest, Romania; ^5^ Rheumatology Clinic, St Mary Clinical Hospital, Carol Davila University of Medicine and Pharmacy, Bucharest, Romania; ^6^ St Bagdasar General Surgery Clinic, Carol Davila University of Medicine and Pharmacy, Bucharest, Romania; ^7^ Prof. I. Juvara General Surgery Clinic, Dr. I. Cantacuzino Clinical Hospital, Bucharest, Romania; ^8^ Department of Obstetrics and Gynecology, Polizu Clinical Hospital, Carol Davila University of Medicine and Pharmacy, Bucharest, Romania

**Keywords:** colorectal cancer, postoperative complications, anastomotic leakage, artificial intelligence, improved surgical management

## Abstract

**Introduction:**

Colorectal cancer stands as a predominant cause of cancer-related mortality worldwide. Despite progressive strides in surgical methodologies, the specter of postoperative complications is very large, significantly impacting both morbidity and mortality rates. This review aims to meticulously examine existing scholarly works to gauge the prevalence, severity, and therapeutic approaches to postoperative complications arising from colorectal cancer surgeries.

**Methods:**

Employing a systematic approach, this study reviewed 135 peer-reviewed publications from the period of 2000–2023. The corpus was organized into categories reflective of the postoperative complications discussed: anastomotic leakage, port-site metastases, small bowel adhesions and obstructions, thrombosis, ileus, postoperative infections, urinary dysfunctions, and cardiovascular dysfunctions. Advanced artificial intelligence tools were leveraged for in-depth literature searches and semantic analyses to pinpoint research lacunae.

**Results:**

The analysis revealed that anastomotic leakage and postoperative infections garnered the majority of academic focus, representing 35% and 25% of the studies, respectively. Conversely, port-site metastases and cardiovascular dysfunctions were less frequently examined, accounting for merely 5% and 3% of the literature. The reviewed studies indicate a disparity in the reported prevalence rates of each complication, oscillating between 3% and 20%. Furthermore, the review identified a dearth of evidence-based management protocols, underscored by a pronounced heterogeneity in treatment guidelines.

**Conclusions:**

The literature is replete with analyses on anastomotic leakage and postoperative infections; however, there exists a glaring scarcity of exhaustive research on other postoperative complications. This review emphasizes the pressing need for uniform treatment guidelines and spotlights areas in dire need of further research, aiming at the comprehensive enhancement of patient outcomes following colorectal cancer surgery.

## 1 Introduction

Cancer persistently ranks as a leading cause of mortality worldwide, with recent statistics indicating that in 112 out of 183 countries (61.2%), it is the primary or secondary cause of death among individuals under 70 years old (1). Among cancers, colorectal cancer prominently stands in the top four for both incidence and mortality rates in developed nations, with male/female incidence rates of 29/20 and mortality rates of 13.1/8.4 per 100,000 individuals, respectively. These figures are similarly high, though slightly lower, in developing and underdeveloped countries (1). The treatment strategy for colorectal cancer, particularly in its early and intermediate stages, predominantly involves surgical interventions. These range from local resections to more extensive surgeries such as partial or total colectomy, and, for cases involving liver metastases, hepatic resection (2, 3). The incidence of postoperative complications is influenced by a myriad of factors, including but not limited to the patient's overall health status, comorbid conditions, age, the stage of cancer, the surgical technique employed, and the surgical team's expertise (4). Despite the critical nature of these factors, there remains a gap in comprehensive understanding regarding their interplay and impact on post-surgical complications, as well as on effective management strategies. This gap underlines the necessity for evidence-based guidelines aimed at reducing the occurrence of such complications. In response, this study embarks on a systematic review following the 2020 PRISMA guidelines (5), with the objectives of identifying factors associated with the development of postoperative complications in colorectal cancer surgery, assessing the prevalence of each identified factor, and evaluating their significance in the occurrence of postoperative complications. Additionally, this review aims to compare the efficacy of various management strategies and pinpoint areas lacking in previous research for future exploration.

## 2 Material and methods

A systematic search was carried out for research papers published from January 2000 to May 2023, utilizing the MEDLINE and PubMed databases. Initially, we used ChatGPT 4.0 to directly identify relevant materials. However, we found that the application was unable to correctly identify bibliographic references, so we used it to generate query scripts for database searches based on predefined terms by the authors. We compared the results obtained with those generated by manually entering the search and selection criteria for scientific articles. We found that using ChatGPT generated more consistent results (publication lists) than those obtained through manual input, and we used these results. The first batch of terms searched included “complications”, “risk factors”, “colorectal surgery”, and “colorectal resection”. The ChatGPT4 application was also employed to unearth potentially relevant materials, which were subsequently manually verified for authenticity and compliance by checking the DOI against the established criteria. This initial search phase led to the identification of a second set of terms related to specific complications encountered in colorectal cancer surgeries, including “adhesions and obstructions of the small intestine”, “thrombosis”, “ileus”, “infection”, “urinary dysfunction”, “colonic ischemia”, “cardiovascular dysfunction”, “anastomotic dehiscence”, and “port-site metastases”. A follow-up search using these terms was performed in the same databases, with an additional focus on mortality post-surgical intervention, using the Failure to Rescue (FTR) index, defined as all-cause mortality at 30 days post-complication diagnosis. Only papers published in English and related to human medicine were selected. The search syntax employed was as follows: (((complications[Title/Abstract] OR “Complications"[Mesh]) AND (risk factors[Title/Abstract] OR “Risk Factors"[Mesh]) AND (colorectal surgery[Title/Abstract] OR “Colorectal Surgery"[Mesh]) AND (colorectal resection[Title/Abstract] OR “Colectomy"[Mesh])) AND ((adhesions[Title/Abstract] OR “Intestinal Obstruction"[Mesh]) OR (thrombosis[Title/Abstract] OR “Thrombosis"[Mesh]) OR (ileus[Title/Abstract] OR “Ileus"[Mesh]) OR (infection[Title/Abstract] OR “Infection"[Mesh]) OR (urinary dysfunction[Title/Abstract] OR “Urinary Bladder Diseases"[Mesh]) OR (colonic ischemia[Title/Abstract] OR “Colonic Diseases"[Mesh]) OR (cardiovascular dysfunction[Title/Abstract] OR “Cardiovascular Diseases"[Mesh]) OR (anastomotic dehiscence[Title/Abstract] OR “Anastomotic Leak"[Mesh]) OR (port-site metastases[Title/Abstract] OR “Surgical Wound"[Mesh])) AND (“2000"[Date—Publication]: “3000"[Date—Publication]) AND [“English"(Language)] conducted on May 13, 2023. This comprehensive and methodical search process, including manual verification and the employment of AI tools for search optimization, culminated in the selection of 135 articles. These articles were rigorously reviewed for relevance, citation count, and potential alignment with the initial search criteria. This led to a refined dataset of 98 pertinent publications, ready for in-depth analysis and review. The vast majority of the selected articles were published in the last 10 years, so we consider that the data analyzed are comparable from case to case without the involvement of a historical bias.

## 3 Results

**Figure 1:**
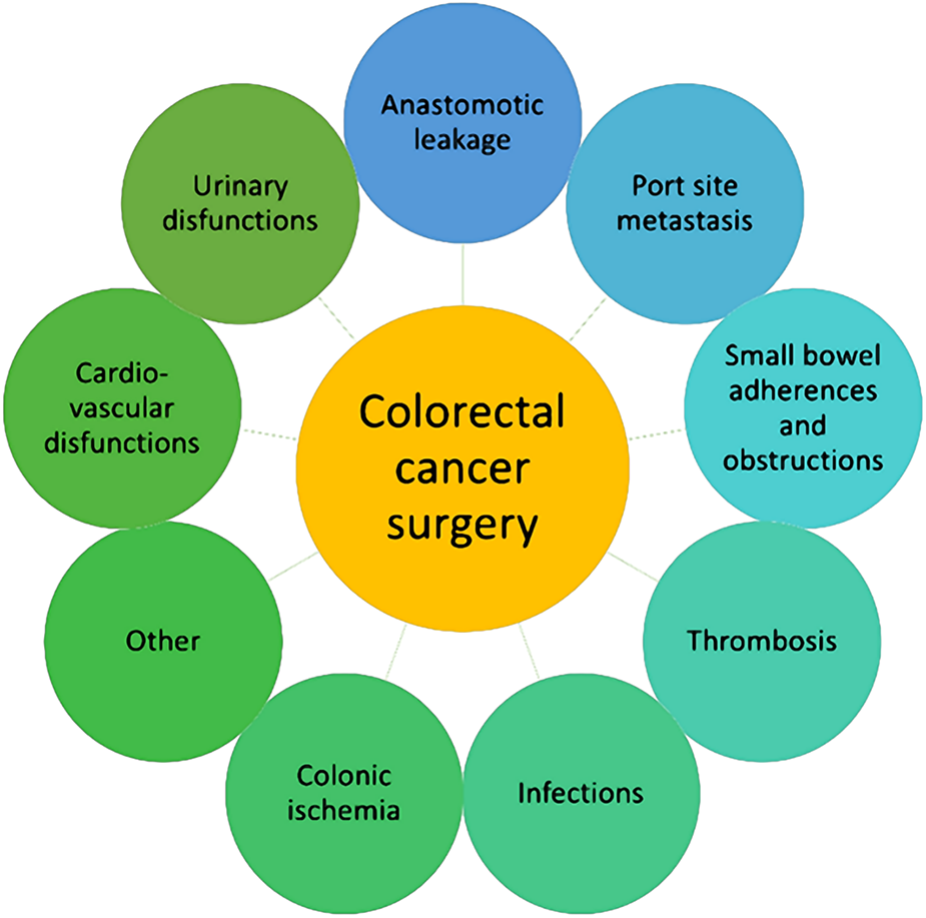
The most commonly reported complications of colorectal cancer surgery.

The comprehensive review of literature published between 2000 and 2023 identified eight predominant types of postoperative complications: anastomotic dehiscence, port-site metastases, adhesions and obstructions of the small intestine, thrombosis, ileus, postoperative infection, urinary dysfunction, and cardiovascular dysfunction (Figure 1). These complications, which are elaborated upon in the following sections, represent the focal points of this systematic review, underlining the complex nature of postoperative care in colorectal cancer surgery and highlighting areas in need of further research and guideline development.

### 3.1 Anastomotic dehiscence

Anastomotic dehiscence as a postoperative complication in colorectal cancer was discussed in 19 articles. For instance, in a study addressing the issue of surgical re-intervention after major complications of the initial intervention in a sample of 14,290 patients who underwent surgery for colorectal cancer, Grönroos-Korhonen et al. (6) found that the re-intervention rate within the first 30 days post-surgery was 5.8%, significantly higher in emergency interventions than in elective ones. The Failure to Rescue (FTR) rate was 17.4%, with a higher rate also evident in emergency interventions compared to elective ones: 27.1% vs. 8%, respectively; *p* < 0.001. Anastomotic dehiscence was the main cause of re-intervention (36.6% of cases with complications). The authors identified a higher FTR level with advancing age, a high Comprehensive Complication Index (CCI), an increased number of previous surgeries, the presence of malnutrition, and the use of anticoagulants. For patients aged over 80 years old, FTR was 20 times higher compared to those under 50 years old. The authors also highlighted the role of CCI in this difference, as the median CCI for those over 80 years old was 6, while for those under this age, it was 1. Moreover, in a study on patients rapidly discharged post-operatively, Depalma et al. (7) evaluated the results of an enhanced postoperative recovery program in patients aged over 80 years old who had undergone laparoscopic surgery. Upon evaluating 162 consecutive cases, the authors identified the presence of anastomotic dehiscence in only 1.8% of cases. In another relevant study on a total of 280 patients, Peters et al. (8) assessed the outcomes of a perioperative enteral nutrition program enriched with lipids compared to standard care. The results did not provide statistically robust support for either of the two options: 9% of patients in the intervention group and 8% in the control group presented with anastomotic dehiscence. The occurrence of anastomotic dehiscence was also investigated in patients with previous resection for rectal cancer by Hultberg et al. (9). After evaluating 1,495 patients, of whom 27% received NSAIDs for at least two days in the first postoperative week, anastomotic dehiscence was identified in 11% of those who received NSAIDs compared to 14% of those who did not (after adjusting for confounders, an odds ratio of 0.65–1.20 with a 95% confidence interval was calculated). In addition, no difference was identified between the groups using selective COX2 NSAIDs vs. non-selective ones. Furthermore, in a study that evaluated the risk of dehiscence in conjunction with the presence of aortic calcifications, Eveno et al. (10) found a positive correlation between dehiscence and aortic calcifications, albeit in a small number of cases included (*N* = 60). Additionally, in a study on the risk factors for prolonged ileus in a large group of 27,560 patients, Moghadamyeghaneh et al. (11) found that 12.7% of the patients experienced prolonged ileus; of these, there was a significantly higher proportion of patients with prolonged ileus and anastomotic dehiscence (12%) compared to the group without dehiscence (2.4%). Finally, in a meta-analysis evaluating data from 23 studies comprising a total of 110,272 patients, Pommergaard et al. (12) determined the following three potential determinants of anastomotic dehiscence: low rectal anastomosis (OR = 3.26), male sex (OR = 1.48), and preoperative irradiation (OR = 1.65).

### 3.2 Port-site metastases

Port-site metastases as a postoperative complication in colorectal cancer were discussed in two articles. Although, as mentioned in Section 2, these studies did not meet our performance criteria, they concluded that in conjunction with laparoscopic surgery and new drug therapies, the associated risk of port-site metastases was reported to be significantly reduced.

### 3.3 Adhesions and obstructions of the small intestine

Adhesions and obstructions of the small intestine as postoperative complications in colorectal cancer were highlighted in four articles. However, as mentioned in Section 2, all four articles approached this subject from a perspective that was not the focus of this review.

### 3.4 Thrombosis

Thrombosis as a postoperative complication in colorectal cancer was identified in 4 articles in our database. For instance, Moghadamyeghaneh et al. (13) evaluated a database consisting of 108,898 patients who underwent surgery for colorectal cancer. Among these patients, 15.6% presented with a moderate level of preoperative hypoalbuminemia (serum albumin between 3 and 3.5 g/dl). In these patients, an increased rate of deep vein thrombosis was identified (adjusted OR = 1.64). The authors concluded that for each unit decrease in albumin below the normal value, there was a 24% increase in morbidity and a 49% increase in mortality.

### 3.5 Ileus

Ileus was discussed in the context of surgical interventions for colorectal cancer in 19 publications. Morimoto et al. (14) evaluated a total of 417 patients with visceral obesity (VO) as identified based on digitized image analysis. In this study, VO was defined if the area of visceral fat was at least 100 cm^2^. The results revealed ileus in 4.3% of cases. Significant risk factors included VO (OR = 7.9, 95% CI 1.9–32.1, *p* = .004), open surgery (OR = 6.4, 95% CI 1.6–26.7, *p* = .010), and pelvic or intra-abdominal abscess (OR = 11.0, 95% CI 1.1–110.2, *p* = .041). In another relevant study on a large sample of 4,205 patients, Grass et al. (15) identified 377 (9%) patients with postoperative ileus. Ileus occurred on average around day 4 postoperatively (interquartile range, 2–5). Intraoperatively, those patients who later developed ileus received a significantly higher volume of fluids compared to those who did not develop this postoperative complication (3.2 ± 2.6 L vs. 2.5 ± 1.7 L, *p* < 0.001). The results of subsequent analyses identified the following risk factors associated with the occurrence of ileus: perfusion with 3 L on the first postoperative day (OR = 1.65, 95% CI 1.13–2.41; *p* = 0.009), weight gain of over 2.5 kg on postoperative day 2 (OR = 1.49, 95% CI 1.01–2.21; *p* = 0.048), and postoperative complications (OR = 2.00, 95% CI 1.39–2.90; *p* < 0.001). Furthermore, in a randomized study on 280 patients, Peters et al. (8) evaluated the role of enteral nutrition enriched with lipids in preventing postoperative complications. The results showed that postoperative ileus occurred in 28% of patients in the intervention group compared to 22% in the control group (risk ratio RR = 1.09, 95% CI 0.95–1.25; *p* = 0.24). Considering that the difference did not reach statistical significance, the authors concluded that enteral nutrition did not impact the risk of developing postoperative ileus. Another factor studied in relation to the development of postoperative ileus is racial background. In an analysis of a large database of 28,283 patients, Mulhern et al. (16) established that, after data adjustment, individuals of Asian origin have a lower risk of developing postoperative ileus (OR = 0.8, 95% CI 0.66–0.98, *p* < 0.001). In unadjusted data, individuals of Asian origin, compared to other racial/ethnic groups, were more likely to have normal weight, not smoke, and have a lower American Society of Anesthesiologists score of 1 or 2 (*p* < 0.001). Koller et al. (17), evaluating the risk of postoperative complications in relation to bowel preparation, analyzed data from 32,359 patients. A significant reduction in the rate of ileus occurrence was observed in the groups that received bowel preparation, with the OA and MBP + OA groups showing a significant reduction in the rate of ileus occurrence, as well as a shorter hospital stay. An increased risk of postoperative ileus was also reported by Lee and colleagues (18) in a study on 3,188 patients who underwent laparoscopic surgery for colorectal cancer. Among the patients in this study, 18.6% had a history of previous surgical intervention prior to the laparoscopic procedure in question. The analyses identified an increased risk of postoperative ileus (5.5% vs. 2.0%, *p* = 0.008) in patients with a history of major surgical intervention. Likewise, in a study previously reviewed in Section 3.1, Moghadamyeghaneh et al. (11) found that ileocolonic anastomosis was associated with a significantly higher rate of ileus compared to colorectal anastomosis (15% vs. 11.5%, adjusted OR = 1.25, *p* < 0.01). Furthermore, a higher risk of ileus was identified in conjunction with preoperative sepsis (adjusted OR = 1.63, *p* < 0.01), disseminated cancer (adjusted OR = 1.24, *p* = 0.01), and chronic obstructive pulmonary disease (adjusted OR = 1.27, *p* = 0.02). A reduced risk of prolonged ileus was also associated with intestinal preparation with oral antibiotics (adjusted OR = 0.77, *p* < 0.01) and laparoscopic surgery (adjusted OR = 0.51, *p* < 0.01). Finally, in an analysis of data from 33,033 patients who had undergone surgery for colorectal cancer, Kelly et al. (19) found that patients with early discharge (hospital stay duration of a maximum of 3 days) were more prone to being readmitted for ileus (OR = 1.8, *p* = 0.001).

### 3.6 Postoperative infections

Postoperative infections as a postoperative complication in colorectal cancer were evaluated in 31 articles. For instance, in Kane et al.'s (20) analysis of 21,889 cases, of which 63.2% received postoperative administration of antibiotics associated with mechanical bowel preparation, the group that received the aforementioned intervention against postoperative infections significantly more frequently included male patients, patients with a body mass index (BMI) of 30–39 kg/m^2^, patients with independent functional status, as well as those scheduled for laparoscopic and robotic surgical approaches. Furthermore, in an investigation of the impact of malnutrition on the occurrence of postoperative infection in a sample of 11,357 patients, Lee et al. (21) identified a higher risk of postoperative infection in malnourished individuals compared to those without this condition (6% vs. 2.62%, odds ratio 2.38, 95% confidence interval 2.07–2.73, *p* < 0.001), suggesting that malnutrition is a significant factor that can lead to postoperative infections as a complication in colorectal cancer. Another factor predisposing patients to postoperative infections as a complication in colorectal cancer identified in the reviewed studies was obesity. For instance, in an analysis of 74,891 cases, of which 33.0% were overweight (BMI 25.0–29.9), 19.8% had class I obesity (BMI 30.0–34.9), 8.4% had class II obesity (BMI 35.0–39.9), and 5.5% had class III obesity (BMI≥40.0), Wahl et al. (22) found that the risk of surgical site infection increased with an increase in the patient's obesity class. From obesity class I to III, the risk of infection increased as follows: for class I, odds ratio (OR) = 1.5 [95% confidence interval (CI), 1.4–1.6]; class II: OR = 1.9 (95% CI, 1.7–2.0); and class III: OR = 2.1 (95% CI, 1.9–2.3). The occurrence of postoperative complications, including postoperative infections, was also studied in association with perioperative anemia. Accordingly, in a sample of a total of 326 patients, Liu et al. (23) identified that perioperative anemia, defined as a hemoglobin level below 12 g/dl, is associated with an increased risk of infection (odds ratio [OR] = 2.44, 95% confidence interval [CI] 1.09–5.49). Three further factors linked to the risk of postoperative infections reported in the literature include high inflammatory disease intensity, the need for manual assistance, and obesity. In a study on 419 patients who had undergone surgery, Drosdeck et al. (24) found that the incidence of surgical site infection was 10.3%, and that the risk of developing surgical site infection [odds ratio (OR)] was evaluated as 3.3, 2.2, and 1.06 in high inflammatory disease intensity, the need for manual assistance, and obesity, respectively. In Mulita et al.'s (25) analysis, between November 2019 and February 2021, 141 patients underwent surgery for colorectal cancer at a tertiary hospital. Of these, 69 were males and 72 females, with 18 cases (12.77%) diagnosed with postoperative sepsis. It was found that patients over 65 years old had a significantly higher risk of sepsis (*p* = 0.034). Patients with an ASA score > 2 developed sepsis more frequently (*p* = 0.008), as did those with diabetes (*p* = 0.013) and cardiovascular disease (*p* = 0.009). Anastomotic leakage was the main cause in 3.55% of cases.

### 3.7 Urinary dysfunctions

Urinary dysfunctions as a postoperative complication in colorectal cancer were mentioned in three publications in our database. For instance, in a meta-analysis aiming to define the effectiveness and safety of Enhanced Recovery After Surgery (ERAS), Liu et al. (26) reviewed 14 studies conducted on a total of 5,961 patients. Urinary complications as a postoperative complication in colorectal cancer were predominantly identified in the elderly group, (OR = 1.639, I2 = 37.63%, 95% CI 1.168–2.299, *p* = 0.0043). However, one of the limitations of this meta-analysis is that, although a possible lower age limit of 85 years was mentioned, a clear definition for the term “elderly” was not provided.

### 3.8 Cardiovascular dysfunction

Cardiovascular dysfunction as a postoperative complication in colorectal cancer was analyzed in 16 articles. For instance, in the study previously reviewed in Section 2.7, Liu et al. (26) found that patients in the elderly  = age group showed a higher number of cardiovascular dysfunctions (OR = 3.361, I2 = 57.72%, 95% CI = 1.072–10.542, *p* = 0.0377). Furthermore, in a meta-analysis of 15 studies on complications of laparoscopic and open approaches in colorectal cancer surgery, Fujii et al. (27) found that, among the 1,436 patients in the laparoscopic group (as compared to 1,810 in the open group), cardiovascular complications were reduced in the short-term evaluation (OR = 0.4767, 95% CI 0.2805–0.8101, *p* = 0.0062). However, no statistically significant differences between the two approaches in terms of survival were observed in the long term. Furthermore, evaluating the impact of chronic kidney disease in 708 cases followed for 21–65 months (median = 45 months), Currie et al. (28) concluded that patients with chronic kidney disease were significantly more prone to developing cardiovascular morbidity and 30-day mortality (4.8% in the chronic kidney disease group vs. 2.1% in the group without renal impairment, *p* < 0.001). Finally, in a comparison of the safety of laparoscopic and open surgeries based on the duration of the surgical procedure that included 4,273 patients (18.4% underwent laparoscopic surgery, 11.3% underwent open surgery), Bailey et al. (29) concluded that a surgical duration greater than 3 h did not provide any additional benefits to the patient in terms of mortality or incidence of cardiovascular complications.

## 4 Discussions

Colon cancer is the third most common type of neoplasia and cause of cancer-related death worldwide. Colorectal surgery is very complex, as it frequently associates very important complications and mortality, with reported morbidity rates of up to 35%, highly variable between centers (30–33). This study focused on identifying predictors of postoperative complications following colorectal surgery, reviewing a total of 135 articles published in 2000–2023 on postoperative complications of colorectal cancer. Overall, the number of papers addressing each complication suggested a marked imbalance in attention regarding each one. While for some complications (e.g., prolonged ileus or cardiovascular dysfunction), a significant number of studies and meta-analyses are available (e.g., prolonged ileus or cardiovascular dysfunction), other complications (e.g., small bowel adhesions) had only a limited number of articles, resulting in reduced relevance. The conditions associated with the development of the evaluated complications were generally well highlighted in the reviewed articles, providing novel insight into their hierarchy and potentially allowing for the development of a decision tree to facilitate a safer approach to patients with colorectal cancer. Furthermore, the use of AI proved effective in generating the necessary syntax for querying electronic libraries within a short time frame. Additionally, AI was employed to improve these syntaxes, which further enables the identification of each targeted complication through consecutive and partially overlapping approaches that also covered areas untouched by previous syntaxes.

Based on pre-established performance criteria for the articles to be evaluated, the most frequent complications after colorectal surgery were found to be anastomotic leakage, wound complications, intraabdominal infection/collections/abscesses, intraabdominal bleeding, postoperative ileus and bowel obstruction, ischemic colitis, systemic complications (sepsis and shock, cardiovascular complications, thromboembolism, respiratory failure/respiratory infections such as pneumonia, atelectasis, coagulation abnormalities), port-site metastases, organ injury, such as ureteral lesions during proctectomy. An important percentage of the patients even develop multiple complications (30, 33–38). The commonly reported risk factors for the occurrence of postoperative complications are the number of resected organs, length of the surgery (more than 120 min), the emergent character of the operation, contamination, blood loss, the experience of the surgeon, patient comorbidities, preoperative hypoalbuminemia, ASA level of III or more, male gender, age (more than 70 years), tumor stage (T3-T4), a low or mid localization for the rectal tumors, colorectal surgery other than sigmoid colectomy (30, 34–38). While some of the complications that appear after open/laparoscopic/robotic colorectal surgery are reported following natural orifice specimen extraction surgery as well, some are specific or can be more frequent following such techniques, including intra-abdominal contamination, fecal incontinence, colpitis, vaginal wall ulcerations, rectovaginal fistulas, tumor recurrence at the extraction site or even intraoperative organ lesions (colon or bladder injury) (39).

Anastomotic leakage is the most common as well as the most feared complication as it is life-threatening, associating additional morbidity, significant mortality, high reoperation rates, increased hospitalization time, patient hospital readmission and substantial costs for the healthcare system. Anastomotic leakage rates vary largely between centers, with average reported values of 7.4%–8.7% of the operated cases (from 2 to 7% when reported by experienced surgical teams, and even up to 24% in case of distal rectal cancer (31, 38, 40, 41). The reported mortality in case of anastomotic leakage after colorectal surgery can be as high as 20% and even up to 39%, depending on the reporting center, explaining the current preoccupation with preventive strategies against the occurrence of anastomotic leakage (6, 42). At the same time, the anastomotic leakage is associated with an increased risk of cancer recurrence, with a significant negative impact on the free-disease and overall survival of the operated-on patients (40, 43). Another concern in regard to the occurrence of anastomotic leakage is the difficulty of therapeutically managing such a complication, with frequent requirements of a second operation (30). In this regard, three degrees of severity of anastomotic leakage have been described. Grades A and B of anastomotic leakage can be managed conservatively, while grade C requires reoperation (34). The therapeutic management of A and B grades includes antibiotics and various forms of drainage (tubal drainage, CT-guided drainage of the collection/abscesses, endoluminal vacuum-assisted therapy with endoscopically leak-site-placed polyurethane sponges, transanal drainage), depending on the site and severity of the leakage, patient status and tumor stage and type of performed surgery. Instead, for the C-grade anastomotic leak, a reoperation is required that is associated with important morbidity and mortality. The reoperation rates reported in the first 90 days from the initial operation vary considerably between centers, between 7% and 14.3% or even up to 78.3% of the cases, and can frequently be considered under-reported (6, 32, 36, 44).

Therefore, finding predictors for the occurrence of anastomotic leakage after colorectal surgery is essential, being a requisite to identify high-risk patients, where a temporary stoma would be preferable to an anastomosis. There is an important variability in the reported rates of anastomotic leakage occurrence between various centers, between 0% to 16% or even 19.2% (34, 42), a percentage that did not significantly decrease despite the progress in medicine with time.

Until now, the existing studies have highlighted several preoperative, intraoperative and postoperative risk factors associated with anastomotic leakage after colorectal surgery, such as age, increased body mass index, malnutrition, male gender, smoking that is associated with vascular ischemia, alcohol consumption, use of steroids and even of NSAIDS, hypoalbuminemia, hypoproteinemia, patient comorbidities, neoadjuvant therapy, tumor stage, emergency surgery, length of the surgery, classification as “contaminated” at the time of the initial surgery, blood loss with need for transfusion, preoperative radiation, postoperative hyponatremia or hyperglycemia. Frequently, multiple risk factors concur in the occurrence of anastomotic leak, while some authors highlight that the most important risk factor is bowel vascular ischemia at the site of the anastomosis (30, 45, 46). Only a few specified factors are controllable and should be corrected actively. For example, the emergent character of an operation can not be controlled. Approximately half of the emergency surgical procedures are represented by colorectal interventions (6). In this concern, in order to prevent “emergency surgery”, we could intervene by screening and early diagnosis of colorectal cancer. Also, in non-emergent surgery, acknowledging patient comorbidities as risk factors, including atrial fibrillation, cardiac failure, ischemic heart disease and other cardiovascular diseases, chronic obstructive pulmonary disease, dementia, connective tissue diseases, diabetes with ASA grade higher than III, and treating them as much as possible becomes essential for the prevention, diminishing or decreasing the severity of the important postoperative complications. Atrial fibrillation is associated with the risk of thromboembolism, further arrhythmias, and hemodynamic compromise, as well as postoperative bleeding in the setting of anticoagulation (46).

At the same time, in the case of association of uncontrollable factors, the patient should be regarded as “high risk”, and a stoma would be the safest procedure (42). There are also intraoperative risk factors, including contamination, blood loss with blood pressure changes, increased length of the surgery and low anastomotic site (34, 45) that should be acknowledged and prevented as much as possible. Some authors report that in emergency surgery, left hemicolectomy and rectal surgery are associated with lower anastomotic leakage rates, probably because a stoma is performed more frequently in such cases rather than in the case of right hemicolectomies. In contrast, other authors found that left hemicolectomy is associated with higher anastomotic leak rates than right hemicolectomy (45, 47). Also, for rectal cancer, the laparoscopic approach for low and ultralow was reported to be safer (34). However, other studies have found a higher risk for colorectal laparoscopic interventions (45). At the same time, several postoperative risk factors have been reported as well, such as postoperative hypoalbuminemia, diarrhea, patient comorbidities, mobilization, and physical activity and medication (45, 48). Other authors have reported the requirement for adequate postoperative medication, as certain drugs, including antibiotic prophylaxis, can decrease the risk of anastomotic leakage, while others are considered risk medicines. In this concern, some authors have reported a protective role for the probiotics administered after colorectal surgery (31, 42). Also, the use of anticoagulants appears to be associated with a higher anastomotic leakage risk (49). At the same time, preoperative bowel cleaning (mechanical or oral preparations) to control the gut microbiome appears to be essential (40).

Several intraoperative preventive strategies against the occurrence of anastomotic leakage have been considered up to now, including the use of nickel-titanium rings for anastomoses instead of manual or stapled sutures, anastomoses’ strengthening via adhesives (cyanoacrylate, gelatin sealants) or wrapping (either with momentum or with mesenteric flaps), verifying the oxygenation level that is essential for the anastomosis (via pulse oxymeters and colonic oxygen saturation measurement), as well as the use of fluorescence angiography with indocyanine green (34).

Following colorectal surgery, anastomotic leakage can occur early, within the sixth postoperative day, or later, usually between the seventh and the 12th postoperative day. However, there are also reports of later anastomotic leaks during an interval of 30 postoperative days, after 30 days or even cases of chronic fistulas that are diagnosed with delay during 90 days from the surgical intervention. Almost 24% of the patients develop anastomotic leaks after discharge, while 12% of the patients after 30 days postoperatively. Regardless of the definition, the ethiopathogeny for the early and late anastomotic leaks differs. In fact, the term early and late anastomotic leaks are not clearly defined yet, as other authors consider “early” as within the first 30 days postoperatively, while late is after 30 days postoperatively. Early leaks are related to surgical technical factors, while late anastomotic leaks occur due to healing defects (41, 50–55). The clinical signs of anastomotic leakage include signs of peritonitis/intra-abdominal or pelvic abscesses such as abdominal pain, fever, sepsis, tachycardia, dynamic ileus (absence of bowel peristalsis by the fourth postoperative day, purulent rectal discharge, fecal/gas/purulent drainage or increased tubal drainage (more than 400 ml); diarrhea before the seventh postoperative day, signs of kidney failure. The diagnosis is based on the aspect of the tubal drainage, with fecal, purulent, or gas discharge, clinical signs, radiological imaging (CT, water-soluble enema), as well as on several biological markers from blood and drained peritoneal fluids (36, 42, 45). CRP is an already proven very effective biological marker in predicting the occurrence of anastomotic leakage after colorectal surgery. Normally, the CRP value increases in the first 48 h after the surgical intervention but afterward decreases towards normal. However, maintaining high values or further increases in CRP levels signal postoperative complications, such as anastomotic fistulas. Therefore, normalization of CRP levels after the initial 48 h following colorectal surgery is considered to be a very significant negative predictor for the occurrence of anastomotic leakage. Instead, an increased CRP level on the fourth to the seventh postoperative day indicates an anastomotic fistula's occurrence (45). Along with the CRP levels, other acute phase reactants (inflammatory and oxidative stress markers) are regarded as very effective predictors for anastomotic leaks, such as white cell count, neutrophil count, procalcitonin, as well as the levels of various interleukins and cytokines from blood and peritoneal fluid from the abdominal drains including Il-6, IL-16, IL-21, TNF-alpha (42, 45). For example, increased serum and peritoneal fluid levels of IL-16, IL-21, chemokine C-C motif ligand 8 or monocyte chemotactic protein-2 (CCL8/MCP-2), chemokine C-X-C motif ligand 13 or monocyte chemotactic protein −4 (CCL13/MCP-4), C-X-C motif chemokine 5 or epithelial neutrophil-activating-peptide (CXCL5/ENA-78), leukemia inhibitory factors on the third postoperative day were found to be associated with the occurrence of anastomotic leaks after colorectal surgery. Among the oxidative stress markers, decreased levels of the antioxidant catalase marker and superoxide dismutase, as well as increased levels of malondialdehyde in the peritoneal fluid and patient serum, are useful diagnostic tools for the anastomotic leaks (45). Other authors have proposed plasmatic citrulline levels and ischemia-modified albumin as effective predictors of anastomotic leakage (46).

As high-volume and low-volume centers report similar postoperative complication rates, the capital difference in patient prognostic is in the early diagnostic and adequate and timely treatment of the anastomotic leak. However, in this concern, high-volume centers from high-income regions appear to have better results, with lower failure-to-rescue rates (6).

Postoperative slow return of bowel peristalsis and even ileus due to dysfunctional intestinal peristalsis or adherences are other complications following colorectal surgery, usually seen starting with the fourth day postoperatively. The dysfunctional intestinal peristalsis is generated by the surgical trauma and inflammatory response and is accompanied by pain, nausea/vomiting, and the impossibility of oral intake and significantly increases the risk of anastomotic leakage occurrence, as well as other complications, such as infections and deep vein thrombosis. Risk factors for the occurrence of postoperative ileus are the type and the length of the operation, blood loss, anesthesia, the use of postoperative opioids, patient-related factors such as age, male sex, increased BMI, history of smoking and alcohol consumption, comorbidities such as respiratory or vascular disease, history of previous operations that associated adhesions (30, 34, 36). Paralytic postoperative ileus can be treated via nasogastric decompression and the replacement of narcotics with steroids to control pain (36). Regarding the intestinal obstruction caused by postoperative adhesions, most of the cases are managed conservatively via various means of bowel decompression, but a small percentage of cases will require reoperation, preferably a laparoscopic intervention. The incidence of postoperative adhesions is important, as approximately 10% of colorectal surgical interventions develop bowel obstruction due to adhesions. In order to prevent the occurrence of postoperative adhesions that could lead to bowel obstruction, along with control of the known risk factors as much as possible, various bioresorbable films have been proposed, such as Seprafilm, carboxymethyl cellulose hyaluronate, hyaluronic acid, carboxymethyl cellulose, poly (L-lactide-co-D, L-lactide). Various risk factors have been identified, including a longer duration of the surgical intervention, emergency procedures, dysfunctional stomas, and male gender, while no significant differences appear between laparoscopic and open surgery (34, 36). Instead, laparoscopic colorectal surgery may be associated with port site metastases (34).

Also, up to 28.5% of the patients can develop perioperative organ failure requiring intensive care, associating additional morbidity, increased hospitalization and costs, and failure to rescue with higher mortality (6).

A percentage of the operated cases, of 7% up to 14.3%, will develop complications requiring reoperation, especially in the patients where emergency operation was initially performed. Although the reintervention rate has been suggested to become a marker for the surgeon's performance, the reported reoperation rates are important and sometimes are even under-reported. The most frequent causes of reoperation are anastomotic dehiscences, bowel obstruction, bleeding, and fascial rupture (wound dehiscence). Postoperative bleeding is rare, and its occurrence depends on the patient's comorbidities, clotting system, and the type of surgery that was performed. The second intervention is an emergency in approximately one-third of the patients. However, a reintervention associates important morbidity organ failure, failure to rescue, and significant mortality with a three times higher risk of death within 90 days postoperatively compared with those that did not require a reoperation (6, 36, 44).

Therefore, some postoperative complications, including anastomotic leak cases, could be avoidable, while others cannot be prevented. In this regard, an active approach with the early diagnosis of the fistula and effective and timely treatment would make the difference. Preventive strategies should be implemented to diminish the occurrence and severity of such complications. However, such a scenario is only sometimes possible, as many uncontrollable risk factors may intervene. Therefore, the goal is to adequately treat the complications, that is, decrease the failure to rescue rates.

The limitations of this study include our focus on studies published exclusively in English, which may have led to overlooking valuable research findings published in other languages. Another limitation of this study is that, in selecting the article for review, we included studies that focused on cohorts with different characteristics of colorectal cancer in terms of tumor location, disease stage, and surgical techniques. These factors, along with the variance in the experience of surgical centers and the variable quality of the healthcare systems where the evaluations were conducted, highlight possibilities for improvement in the standardized reporting of results. Furthermore, since the evaluated studies exhibited significant differences in identifying factors that may have contributed to different patient outcomes, direct comparisons may be challenging. This review has unlikely left any highly informative elements uncovered and provides a thorough review of the state-of-the-art research on postoperative complications of colorectal cancer.

## 5 Conclusion

This review identified both modifiable (e.g., enhanced postoperative recovery programs) and non-modifiable factors (e.g., age, sex) influencing postoperative outcomes in colorectal cancer surgery. In further research, in order to better understand the extent to which current efforts to reduce postoperative complications in colorectal cancer are justified, it would be necessary to evaluate the relative weight of each factor in the corresponding patients’ outcomes. Doing so would promote the development of a perioperative approach algorithm based on these factors that would ultimately improve the prognosis of patients with colorectal cancer.
